# Structural, electrical, and magnetic study of La-, Eu-, and Er- doped bismuth ferrite nanomaterials obtained by solution combustion synthesis

**DOI:** 10.1038/s41598-021-01983-z

**Published:** 2021-11-23

**Authors:** Angelika Wrzesińska, Alexander Khort, Marcin Witkowski, Jacek Szczytko, Jacek Ryl, Jacek Gurgul, Dmitry S. Kharitonov, Kazimierz Łątka, Tadeusz Szumiata, Aleksandra Wypych-Puszkarz

**Affiliations:** 1 Lodz University of Technology, Zeromskiego 116, 90-924 Lodz, Poland; 2 KTH Royal Institute of Technology, Stockholm, Sweden; 3 National University of Science and Technology “MISIS”, Moscow, Russia; 4 University of Warsaw, Pasteura 1, 02-093 Warsaw, Poland; 5 Gdańsk University of Technology, 11/12 Narutowicza st, 80-233 Gdańsk, Poland; 6 Jerzy Haber Institute of Catalysis and Surface Chemistry, Polish Academy of Sciences, Niezapominajek 8, 30239 Kraków, Poland; 7 Research and Development Center of Technology for Industry, Ludwika Warynskiego 3A, 00645 Warsaw, Poland; 8 Marian Smoluchowski Institute of Physics, Jagiellonian University, Łojasiewicza 11, 30-348 Kraków, Poland; 9 Kazimierz Pulaski University of Technology and Humanities in Radom, Stasieckiego Str. 54, 26-600 Radom, Poland

**Keywords:** Chemical synthesis, Electronic materials, Magnetic materials

## Abstract

In this work, the multiferroic bismuth ferrite materials Bi_0.9_RE_0.1_FeO_3_ doped by rare-earth (RE = La, Eu, and Er) elements were obtained by the solution combustion synthesis. Structure, electrical, and magnetic properties of prepared samples were investigated by X-ray photoelectron spectroscopy, Mössbauer spectroscopy, electrical hysteresis measurement, broadband dielectric spectroscopy, and SQUID magnetometry. All obtained nanomaterials are characterized by spontaneous electrical polarization, which confirmed their ferroelectric properties. Investigation of magnetic properties at 300.0 K and 2.0 K showed that all investigated Bi_0.9_RE_0.1_FeO_3_ ferrites possess significantly higher magnetization in comparison to bismuth ferrites obtained by different methods. The highest saturation magnetisation of 5.161 emu/g at 300.0 K was observed for the BLaFO sample, while at 2.0 K it was 12.07 emu/g for the BErFO sample. Several possible reasons for these phenomena were proposed and discussed.

## Introduction

Bismuth ferrite BiFeO_3_ is a multiferroic that belongs to the group of materials with a perovskite-type ABO_3_ crystal structure. Such crystal structure is formed through a network of corner-linked oxygen octahedra. The unit cell of BiFeO_3_ has rhombohedral (*R*3c) symmetry with lattice parameters: *a* = 5.58 Å and *c* = 13.90 Å^1^. The larger cation (Bi^3+^) occupies the A site in the corner position of the unit cell and mostly defines BiFeO_3_ ferromagnetic polarisation. Smaller cations (Fe^3+^) occupy the B site in the face-centered position and are related to the magnetic properties of BiFeO_3_, while oxygen ions are located in the body-centered positions^2^. Due to the ionic sizes of bismuth and oxygen, the BiFeO_3_ structure does not perfectly match the ideal cubic unit cell. Therefore, the oxygen octahedra are tightened in order to fit into the reduced cell. This leads to the rotation of oxygen octahedra in BiFeO_3_ around the polar [111] axis by the angle of 11–14° and directly affects the value of the Fe–O–Fe bond angle^3^. The magnetic exchange interaction, which is related to the bond angle, and the orbital overlap between iron and oxygen can significantly influence the magnetic ordering temperature and the conductivity of the BiFeO_3_ phase. These structural features lead to simultaneously ferroelectric (Curie temperature T_C_ = 1103 K) and magnetic (Néel temperature T_N_ = 643 K) behaviors of BiFeO_3_ at ambient temperature.

Other important features of the BiFeO_3_ phase are related to its magnetic structure. It adopts a G-type antiferromagnetic ordering, i.e. each Fe^3+^ spin is surrounded by six antiparallel spins of the closest Fe neighbours^4^. However, these spins are not perfectly antiparallel and exhibit weak canting moments due to the local magnetoelectric coupling to the polarization. The existence of canting creates a long-range arrangement of a spin-cycloid of the antiferromagnetically ordered sublattices with a period of ca. 62–64 nm and the propagation vector along the [110] direction^1^. Due to the presence of spin cycloid structure, a larger part of the net magnetic moment is cancelled and the magnetoelectric coupling is inhibited. Nevertheless, canting of the spin structure in BiFeO_3_ leads to antisymmetric spin exchange (Dzyaloshinskii–Moriya interaction), resulting in a presence of relatively small ferromagnetic moment^5–7^. A slightly higher magnetic moment can be observed for BiFeO_3_ nanoparticles, for which there is a considerable contribution of uncompensated ferromagnetic spin alignment at the surface^8,9^.

As a result of their unique properties, for the past 20 years BiFeO_3_ and BiFeO_3_-based materials have been widely explored to be used in a broad range of applications in advanced electronics, such as spintronics and information storage (ferroelectric random access memory (FeRAM)^10^, and in magnetic random access memory (MRAM)^11^), photovoltaic^12^, and sensor devices^13^, hyperthermia treatment^14^, photocatalysis^2,15–17^, and as supercapacitors^18,19^. Unluckily, a number of disadvantages hinder the widespread use of BiFeO_3_-based nanomaterials. Among them are low resistivity, resulting from high leakage current inducing large dielectric loss, small remnant polarization, and weak ferroelectric loop at room temperature. For the above-mentioned effects, the oxygen vacancies and secondary phases that nucleate at grain boundaries are responsible. They are difficult to avoid during synthesis and they require careful attention to processing parameters^20^. Moreover, the volatile nature of Bi^3+^ during synthesis causes reduction of Fe^3+^ to Fe^2+^ and formation of anion and cation vacancies. Applicability of this material is also limited due to the presence of complex incommensurate cycloid magnetic structure, which is responsible for weak magnetoelectric coupling^21^. One of the most efficient ways to improve magnetic and ferroelectric properties is ion substitution that induces lattice distortions and decreases particle size, as well as suppresses cycloid spin structure. A broad range of elements was tested as dopants in the A-site or B-site positions in order to influence the internal structure of BiFeO_3_ and suppress the formation of the secondary phase during synthesis. The rare-earth metals have repeatedly confirmed their positive contribution to the properties of doped BiFeO_3_. Reddy et al.^2^ observed the improvement in photocatalytic efficiency, higher specific capacitance values with increasing La doping (La_*x*_Bi_1-*x*_FeO_3_, *x* = 0.01–0.10) and reaching the highest values of coercivity (315 Oe), and saturation magnetisation (0.83301 emu/g) at 10% of La doping. Hong et al.^22^ reported that 5% doping of trivalent ions Sm^3+^, Gd^3+^, and Y^3+^ in BiFeO_3_ improved ferromagnetism due to modulation of spiral spatial structure. Golda et al.^23^ observed enhancement of dielectric, ferromagnetic and electrochemical properties of Sm-doped BiFeO_3_ (Bi_1-*x*_Sm_*x*_FeO_3_; *x* = 0.05 and *x* = 0.1).

Up to now different synthesis methods were reported for successful obtaining of BiFeO_3_-based materials: RF magnetron sputtering^24^, polymer solution method^25^, solution combustion synthesis (SCS)^26^. The SCS method is based on exploiting a highly exothermic combustion redox reaction between metal nitrates (oxidizer) and organic fuel (reducer), mixed in the solution. The SCS deserves special attention as it is considered to be time and energy-saving and easy to scale up^27–29^. Besides, this method has been successfully used for the synthesis of a broad variety of materials, including metals^30,31^, graphene^32^, simple and complex oxides including BiFeO_3_^33–35^, so far only a few works have described the application of the SCS for rare-earth-doped BiFeO_3_ preparation^36^.

The present study evaluates the effect of a high-load (10%) La, Er, and Eu doping on the crystallographic structure, magnetic and electrical properties of co-doped BiFeO_3_ nanoparticles obtained by the SCS method.

## Materials and methods

### Synthesis procedure

The detailed procedure for the synthesis of examined materials is described in our previous article^36^. Herein we report only the general description of the main steps.

All materials were prepared using the microwave-assisted solution combustion synthesis (SCS) as follows. Bismuth(III)-nitrate pentahydrate (Bi(NO_3_)_3_∙5H_2_O), iron(III)-nitrate nonahydrate (Fe(NO_3_)_3_∙9H_2_O) and one of the rare-earth metal nitrates (La(NO_3_)_3_∙6H_2_O, Eu(NO_3_)_3_∙6H_2_O or Er(NO_3_)_3_∙6H_2_O) were dissolved in an acidic aqueous solution in such ratios to form the final material with stoichiometric formula Bi_0.9_RE_0.1_FeO_3_. In the text of the paper, they are further abbreviated as BFO (BiFeO_3_), BLaFO (Bi_0.9_La_0.1_FeO_3_), BEuFO (Bi_0.9_Eu_0.1_FeO_3_), and BErFO (Bi_0.9_Er_0.1_FeO_3_). The citric acid (CA) was used as a fuel (reducer). The parameter φ, which is the fuel-to-oxidizer molar ratio, for all experiments was kept constant and equal to 1.25. The obtained solution containing all precursors was rapidly dried in a microwave oven (800 W, 2.450 GHz) until a gel and then highly porous foam had formed. The foam was ignited and burned in a preheated muffle furnace at 573 K in air, leading to the formation of a fluffy brown powder. The obtained powder was hand-milled in an agate mortar and annealed in air at 923 K for 30 min with rapid heating and cooling by means of quenching, after which the resulting powder was hand-milled again. It is assumed that in the systems under study Bi^3+^ is substituted by close in size La^3+^ and smaller Eu^3+^ and Er^3+^ ions (${r{\text{Bi}}}_{ion}^{3+}$ = 1.03 Å, ${r{\text{La}}}_{ion}^{3+}$ = 1.03 Å, ${r{\text{Eu}}}_{ion}^{3+}$ = 0.95 Å, and ${r{\text{Er}}}_{ion}^{3+}$ = 0.89 Å, respectively)^37^.

### Characterization

The X-Ray Photoelectron Spectroscopy (XPS) measurements were carried out in a high-resolution mode, with a pass energy of 20 eV. The studies were performed on an Escalab 250Xi from ThermoFisher Scientific. The spectroscope utilizes monochromatic Al Kα X-ray source with a spot diameter of 650 µm. The charge compensation was provided using low-energy electrons and low-energy Ar^+^ ions emission from the flood gun with a final calibration on adventitious carbon C1s peak (284.6 eV) as a reference. Spectral deconvolution was performed with the Avantage software provided by the manufacturer.

Mössbauer spectroscopy studies were done using the ^57^Fe gamma resonance transition. A Mössbauer system that consists of the Janis top-loaded liquid helium cryostat (Janis Research Company, Wilmington, MA 01887 USA) integrated with a conventional constant-acceleration spectrometer (Science Engineering & Education Co. USA) of the Kankeleit type in transmission geometry was used. During measurements, a 100 mCi Mössbauer ^57^Co(Rh) γ-ray source and the absorbers were kept at room temperature. The absorbers were made of fine powdered materials placed in thin-walled (~ 0.1 mm) cylindrical plastic containers. The used absorber thicknesses of about 10–12 mg/cm^2^ were calculated from the optimisation procedure^38^. The resonance 14.4 keV gamma rays (for a given measurement and the energy scale calibration) were detected simultaneously by means of two independent LND Kr/CO_2_ proportional gas counters attached at opposite sides of the driving system. The drive velocity calibration was performed with a second ^57^Co(Rh) source against a standard metallic iron foil at room temperature. The Mössbauer spectra were analysed numerically by fitting a hyperfine parameter distribution (HPD) using the Voigt-line-based method of Rancourt and Ping^39^. In this method, the HPD for a given crystal site corresponding to similar structural, chemical, and magnetic properties is constructed by a sum of Gaussian components for the quadrupole splitting (QS) distributions and, if necessary, the magnetic hyperfine field B_hf_ distributions. The isomer shift (IS) can be linearly coupled to the primary hyperfine parameters (QS, B_hf_).

For electrical measurement, all powders were hydraulically pressed into a pellet shape by applying the force of 4000 kg for 5 min. Calculated filling ratio of the measured palletized samples is: BFO = 50%, BLaFO = 55%, BEuFO = 49%, and for BErFO = 58%, thus these pellets are composed from ceramic and the air. Additionally, all pelletized samples were dried at 363 K in vacuum to eliminate the influence of humidity. An EDWARDS Auto 306 vacuum evaporator was used for deposition of 150 nm-thick gold electrodes onto both sides of the pellets to provide good contact between the sample and external electrodes during electrical measurements.

The electrical polarization loops of pelletized samples were recorded at room temperature with modified Sawyer–Tower circuit using a sinusoidal signal at 1 kHz frequency.

The dielectric response measurements were carried out using a Novocontrol GmbH Concept 80 broadband dielectric spectrometer equipped with a Quatro Cryosystem in the 10^–1^ ÷ 10^6^ Hz frequency range and the temperature interval from 133 to 473 K with 10 K step.

The magnetic properties were measured using a Quantum Design MPMS-7 SQUID magnetometer. Full hysteresis loops were recorded up to a maximum field of 70 kOe at two temperatures: 300.0 K and 2.0 K. Solid samples were encapsulated in a Parafilm^®^ M envelope, the mass of the sample was determined using a Sartorius SE2 ultramicroweight. The diamagnetic contribution from the envelope to the overall magnetisation was subtracted from databasing on the appropriately scaled magnetisation of the reference sample. Data analysis was carried out using the method of Corbellini et al.^40^, consisting of fitting the Voigt curve to the differentiated halves of the hysteresis curve. However, due to sparse number of experimental points at the low magnetic fields, the fit did not resemble the true nature of the samples. Thus, paramagnetic contributions were subtracted from the hysteresis loop using fitting of sum of Langevin-type curves. The procedure is elaborated in the Supplementary Information. Temperature-dependent magnetisation curves were registered in zero-field cooling/field cooling (ZFC/FC) experiments at 100.00 Oe. The temperature was swept between 310.0 and 2.0 K.

## Results and discussion

### Structural properties and composition

A detailed description of the synthesis procedure, analysis of crystalline phases, and morphology of bismuth ferrite doped with lanthanum, europium, or erbium are presented in our previous work^36^. Herein, we have only provided a short description of the obtained data for a better understanding of our further results. The XRD patterns of the experimental samples are shown in Supplementary Information (Fig. S1). All powders have a fine microstructure with aggregated grains of different morphology^36^, characteristic for SCS-obtained materials^28^. The EDX analysis showed the elemental composition of nanopowders close to the stoichiometric one. According to an analysis of XRD data, synthesized materials are nanocrystalline powders of Bi_0.9_RE_0.1_FeO_3_ crystal phase with traces of Bi_2_Fe_4_O_9_ and Bi_2_O_3_ by-phases. Nevertheless it should be underlined that all invastigated compounds exhibit content of the main phase, equal or higher than 90%^36^. The calculated average crystallite sizes of the main phases are 17 nm, 28 nm, 18 nm, and 18 nm for the neat BFO, BLaFO, BEuFO, and BErFO, respectively. Previously we found^36^, that the high-load doping of BFO with rare-earth ions leads to the distortion of its R3c crystal structure to an extent, which depends on the difference of ionic radii between the bismuth ion and corresponding rare-earth ion. In our case, the degree of distortion increases in a row BFO > BLaFO > BEuFO. Doping by Er^3+^ leads to a distortion of the crystal structure in a degree, which is high enough to promote the formation of two separate BiFeO_3_ phases: low-temperature rhombohedral R3c and orthorhombic Pbnm, metastable at room temperature.

#### XPS analysis

**Figure 1:**
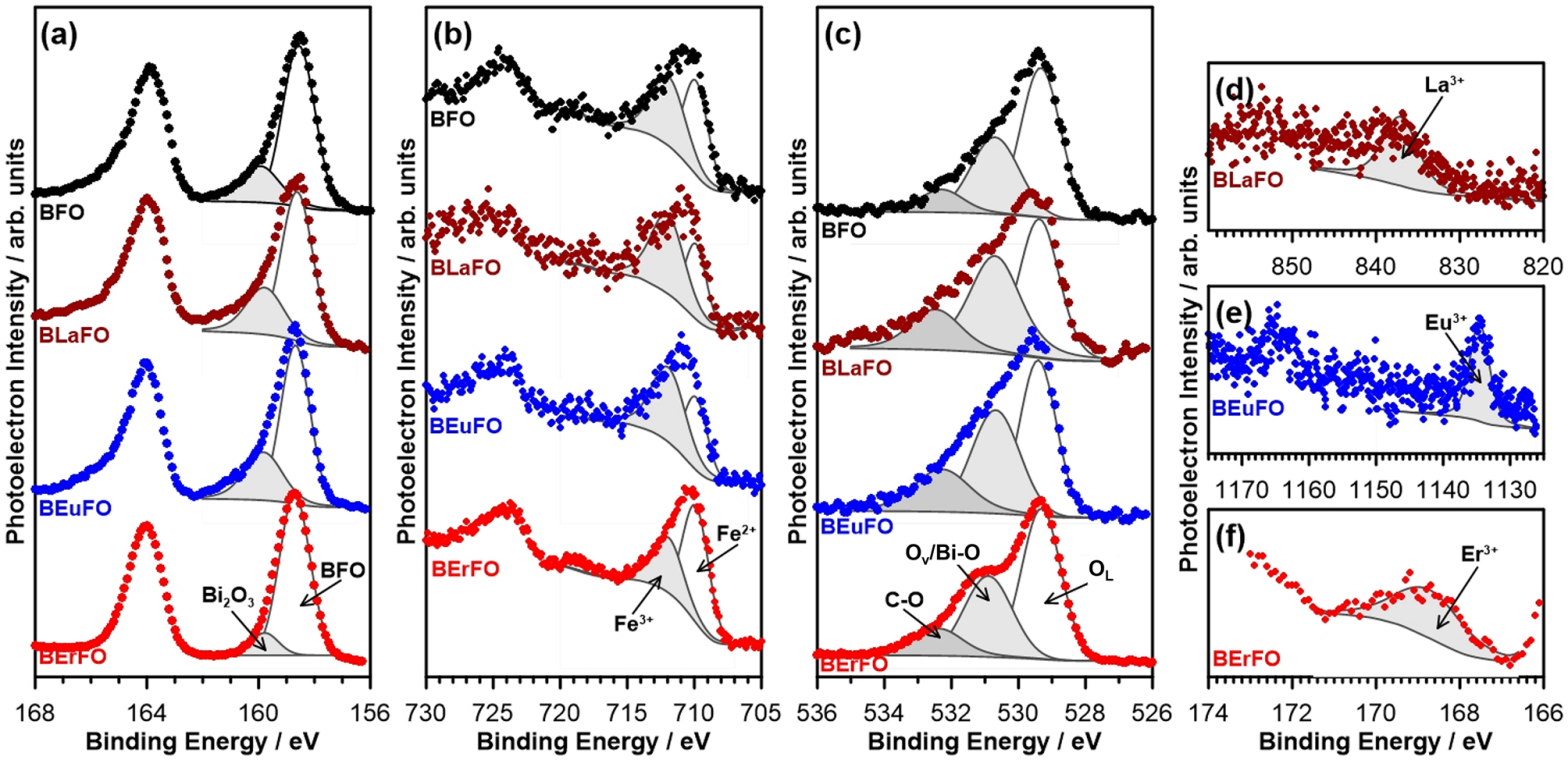
Deconvoluted high-resolution XPS spectra of the experimental samples in binding energy ranges of (**a**) Bi 4f, (**b**) Fe 2p, (**c**) O 1s, (**d**) La 3d_5/2_, (**e**) Eu 3d, (**f**) Er 4p_3/2_.

To study the near-to surface compositions of the experimental samples, high-resolution XPS spectra were recorded for each investigated sample in the Bi 4f, Fe 2p, O 1s, La 3d, Eu 3d, and Er 4p binding energy (BE) range (Fig. 1). The results of the peaks deconvolution are summarized in Table 1. Figure 1a shows the comparison of the XPS spectra recorded in the Bi 4f BE range for each investigated sample with superimposed Bi 4f_7/2_ peaks used for spectral deconvolution. Each spectrum is composed of two peak doublets, with Bi 4f_7/2_ peaks located at 158.7 and 159.8 eV, respectively. The first and dominant of the two components is ascribed to bismuth in the BiFeO_3_ phase, while the latter one, may correspond to Bi_2_O_3_ oxide^41–43^.

**Table 1:** The share of various chemical states of Bi, Fe, O, and rare-earth additives, based on high-resolution XPS analysis and spectral deconvolution.

	Bi4f_7/2_		Fe2p_3/2_		O1s		Additive
	BFO	Bi_2_O_3_	Fe^2+^	Fe^3+^	Fe–O	Bi-O	[Er/Eu/La]^3+^
BE/eV	158.7	159.8	709.9	711.9	529.5	530.7	
BFO	18.6	5.7	4.3	4.4	41.2	25.8	–
BLaFO	16.6	7.0	2.8	4.3	34.0	34.4	0.9
BEuFO	17.4	7.5	3.5	5.2	35.0	30.5	0.9
BErFO	22.9	2.9	3.7	2.1	41.0	26.9	0.5

Results in at.%.

The quantitative assessment of BiFeO_3_:Bi_2_O_3_ ratio, based on Bi 4f_7/2_ peak analysis, reveals that bismuth oxide share does not exceed 23% for undoped BFO, 30% for BLaFO and BEuFO, and 11% for BErFO. As XPS shows near to surface (5–10 nm deep) composition, while XRD could collect data up to 1 µm from surface, the difference between bismuth oxide phase content measured by these two techniques suggests that the concentration of Bi_2_O_3_ is higher near to the surface regions. The observed phenomenon could be related to the higher influence of external factors, like atmosphere, moisture, and temperature gradient during synthesis and quenching, onto the layers of nanoparticles close to their surface. The Fe 2p signal from iron (Fig. 1b) also comes in two peak doublets, where Fe 2p_3/2_ peak corresponding to Fe^2+^ is located at 709.9 eV and Fe^3+^ at 711.9 eV^43,44^, with nearly equal stoichiometry for undoped BFO material. A slightly higher Fe^3+^ share was then recorded for both BEuFO and BLaFO samples, while the BErFO sample is characterized by a higher amount of lower valence Fe^2+^. The peaks for each additive are presented in Fig. 1d–f. Based on the XPS analysis and presence of La 3d_5/2_ peak at 837.1 eV, lanthanum is present within BLaFO as La^3+^, presumably in the form of La_2_O_3_^45^ with a share of approx. 0.9 at.%. A similar observation is drawn for BEuFO, where europium is revealed as Eu^3+^ (Eu 3d_5/2_ at 1134.6 eV)^46^. The analysis of erbium was carried out within Er 4p_3/2_ BE range revealed a slightly smaller share of the element (0.5%), identified as Er^3+^^47,48^.

The spectra recorded in the O 1s BE range and shown in Fig. 1c reveal three XPS peaks. The first component, located at 529.5 eV, is characteristic of Fe–O interaction in Fe–O octahedron^49^ and corresponds to the O 1s core spectrum of bismuth ferrite lattice (O_L_)^50,51^. The peak at 530.7 eV is generally attributed to the chemisorbed oxygen caused by oxygen vacancies (O_v_)^50,51^ or to Bi–O bonds in the BFO phase^42,49^. The low-intensity peak at 532.5 eV reveals C–O interaction. This signal could originate both from small traces of functionalized carbon, left in the samples as a result of incomplete fuel burning out^52^, and CO_2_ molecules, directly absorbed from the air. As the amount of carbon-containing phase is insignificant (not exceeding 7 at.% for each sample), its possible influence was excluded from consideration during further analyses. It is noticeable, that doping with rare-earth elements leads to the development of the peak located at 530.7 eV, implying either a higher share of Bi–O interaction or the appearance of additional oxygen vacancies.

Based on the analysis of the presented XPS results we can conclude that each doped BFO sample reveals the altered chemistry of both Bi and Fe elements, where dopant addition tends to result in the appearance of more oxidized species with Eu and La doping, but more reduced species in the case of erbium. The combination of the influence of rare-earth doping elements with high-intense synthesis and post-synthesis temperature treatment conditions lead to a BFO phase degradation and split into near-to-surface regions of nanomaterials. However, we suppose, such a structure is not replicated in the bulk material. This leads to a formation of the inhomogeneous structure, with BFO-rich inner core, and inhomogeneous outer layers, which consist of a mixture of oxides and non-stoichiometric BFO phases. The interaction of different layers, boundaries, and phases in the described complex structure could influence the properties of the BREFO nanopowders changing their electrical and magnetic surface- and bulk-related characteristics, as described in the text below.

#### ^57^Fe Mössbauer spectroscopy

**Figure 2:**
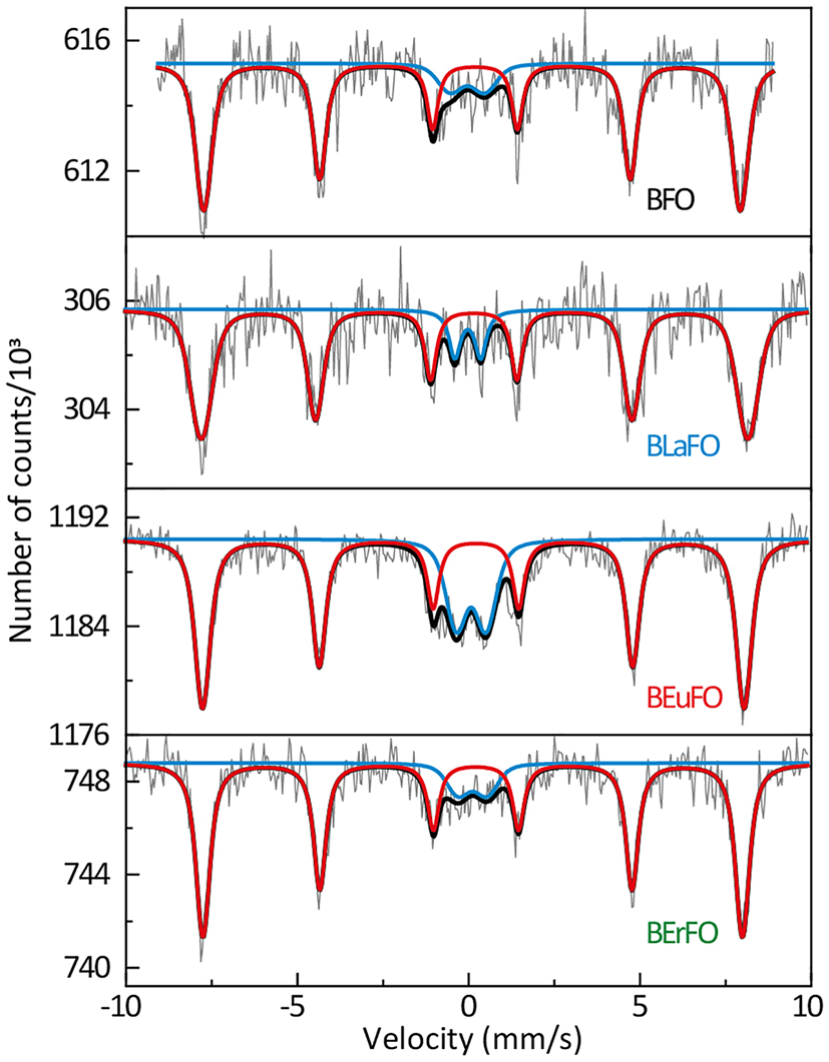
Room temperature ^57^Fe Mössbauer spectra of BiFeO_3_ and BiREFeO_3_.

^57^Fe Mössbauer spectra of the samples, obtained at room temperature, are presented in Fig. 2. The hyperfine parameters of each component and their relative intensities received from the detailed numerical analysis of all spectra are listed in Table 2.

**Table 2:** The hyperfine parameters obtained from the Mössbauer spectroscopy measurements at room temperature (IS—isomer shift, QS—quadrupole splitting, B_hf_—magnetic hyperfine field).

Sample	IS (mm s^−1^)	QS (mm s^-1^)	B_hf_ (T)	Area (%)	HWHM (mm/s)	Components
BFO	0.09	1.00	–	11	0.19	Fe^3+^ PM impurity
	0.27	− 0.04	48.6(9)	89		Fe^3+^ magn. phase
BEuFO	0.20	0.87	–	24	0.20	Fe^3+^ PM impurity
	0.30	− 0.04	49.0(7)	76		Fe^3+^ magn. phase
BErFO	0.24	0.84	–	11	0.20	Fe^3+^ PM impurity
	0.29	− 0.05	48.8(6)	89		Fe^3+^ magn. phase
BLaFO	0.10	0.75	–	10	0.19	Fe^3+^ PM impurity
	0.29	0.01	49.5(1.4)	90		Fe^3+^ magn. phase

*PM* paramagnetic, *HWHM* Lorentzian linewidth.

All recorded spectra have poor signal-to-noise ratio because of the very high absorption coefficient of bismuth for 14.4 keV gamma rays^53^, and interpretation of the results due to poor statistics should be taken with some caution. The room temperature spectra are close to symmetrical, however, the resonant lines are broadened (Table 2) relative to the instrumental linewidths of the calibration spectra.

Such broadening is caused by a distribution of QS, which indicates that not all crystallographic Fe-positions in the rhombohedral crystalline structure of BiFeO_3_ are completely equivalent. Moreover, the spectral asymmetry in BiFeO_3_ could also be caused by an incommensurate spin cycloid structure^54–57^.

All measured spectra are superposition of two components: (i) magnetic sextet related to perovskite-type structure of BiFeO_3_ (BiREFeO_3_) main phase (marked in red) and (ii) quadrupole doublet assigned to paramagnetic Bi_2_Fe_4_O_9_ impurity phase (marked in blue)^56^. The isomer shift of the main component is close to 0.3 mm/s, which is characteristic for high spin Fe^3+^. The magnetic hyperfine field of BiFeO_3_ is very close to the value reported by Palewicz et al.^58^. There is no significant change in the average magnetic hyperfine field (B_hf_) values between Mössbauer spectra of BiFeO_3_ and rare-earth-doped BFO. This could indicate that the doping with the rare-earth elements leads to the replacement of bismuth in the A site of BiFeO_3_ crystal cell, and the number of magnetic nearest neighbors of Fe^3+^ remains the same^56^. It is worth noting that the most stable polymorph of iron oxides α-Fe_2_O_3_, which is a weak ferromagnet at room temperature, if present in our samples, should also result in a magnetic sextet. Thus, the relatively low value of B_hf_ ~ 49 T of the main magnetic phase indicates the absence of ferromagnetic α-Fe_2_O_3_ or any other unreacted iron oxide phases, which would have a much higher hyperfine field up to 51.4 T^59^. On the other hand, a slight admixture of highly dispersed and oxidized magnetite cannot be completely excluded due to its much lower magnetic hyperfine field. The quadrupole splitting of the main component is close to zero, which is characteristic of the localization of Fe^3+^ ions near the center of FeO_6_ octahedra.

The hyperfine interaction parameters of a doublet line are slightly different for all samples. However, they fall into the range of 0.09–0.24 mm/s for IS and 0.75–1.00 mm/s for QS. These values are similar to those reported for Bi_2_Fe_4_O_9_^55,57,60^ and their dispersion, supposedly, originates from the low signal-to-noise ratio. Another possible origin for the quadrupole doublet is the nanoscale structure of BiFeO_3_ grains, which are small enough to become superparamagnetic. For instance, Park et al.^9^ showed similar Mössbauer parameters of the quadrupole doublet for 51 nm crystals of BiFeO_3_. In this case, it is almost impossible to distinguish the oxygen-deficient surrounding of Fe^2+^ (or Fe^3+^) in low spin configuration from superparamagnetic BiFeO_3_ nanoparticles^59^.

### Electrical properties

#### P-E analysis

**Figure 3:**
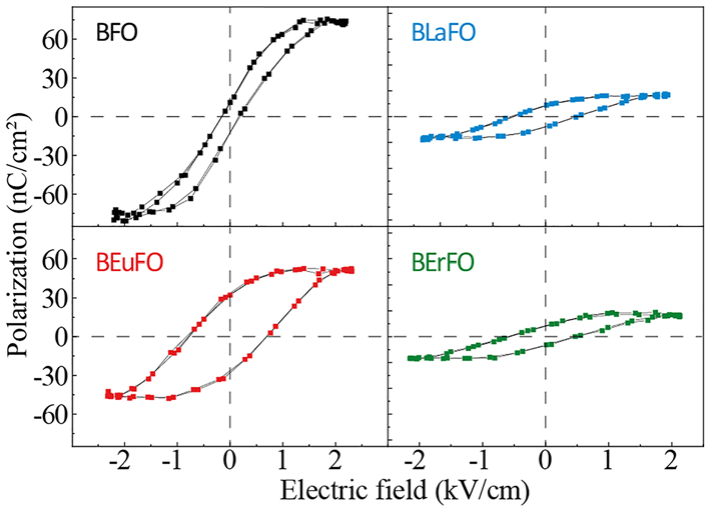
Room temperature ferroelectric (P-E) hysteresis loop of BFO, BLaFO, BEuFO, and BErFO samples.

The correlation between polarization and the applied electric field at room temperature was studied using a modified Sawyer–Tower circuit. Ferroelectric hysteresis (P-E) loops of the neat and doped BiFeO_3_ are shown in Fig. 3.

Crucial parameters of ferroelectrics, such as remnant polarization (P_r_), coercivity (E_c_), and saturation polarization (P_s_) were determined from the obtained hysteresis loops as shown in Table 3.

**Table 3:** The values of coercivity, remnant polarization, and saturation polarization for neat and doped BFO materials.

Parameter	BFO	BLaFO	BEuFO	BErFO
Coercivity (kV/cm)	0.17	0.61	0.75	0.59
Remnant polarization (nC/cm^2^)	11.43	8.20	30.39	7.77
Maximum polarization (saturation polarization) (nC/cm^2^)	76.32	17.27	48.53	16.69

The ferroelectric properties of BFO materials are directly related to the orbital hybridization of Bi^3+^–O^2–^ bonds. The narrow ferroelectric hysteresis loops of ferroelectric materials are characterized by a small amount of dissipated energy (low *E*_c_) in repeatedly reversing polarization cycles.

Generally, it is difficult to obtain a saturated P-E loop for BFO ceramics due to leakage current caused by secondary phases and other defects, which can result in a partial reversal of the polarization. Taking into account samples’ porosity and the fact that investigated pallets are composed from ceramic nanoparticles the measured P-E loops exhibit unsaturated character. The increasing purity of the main BFO crystal phase could also contribute to a decrease in leakage current^61,62^. The experimental samples have relatively low values of remnant polarization and coercive field^63,64^, which indicates the existence of nano-sized ferroelectric domains and confirms the nano-specific character of the properties of studied materials.

However, despite the dominant influence of ferroelectric domains’ size, ferroelectric properties may also be affected by composition, homogeneity, and the presence and distribution of defects in nanomaterials^65^. We suppose the low value of ferroelectric parameters can be associated with the parasitic conductivity on grain boundaries and partially caused by impurity phases. Such conductivity is an intrinsic feature of magnetic compounds and often overlaps with the ferroelectric response of the multiferroic phase^66^. In BFO and BREFO compounds conductivity can be affected and contributed by electron transfer between localized ferric and ferrous ions, i.e. electron hopping between Fe^3+^ and Fe^2+^, morphology/microstructure and hopping of the oxygen vacancies that resembles the dipolar reorientation and results in a dielectric relaxation peak at high temperature^67^. Furthermore, the presence of *P*bnm phase in erbium-doped BFO also leads to a decrease in the ferroelectric response of the samples due to symmetry constraints^68^.

#### Dielectric analysis

**Figure 4:**
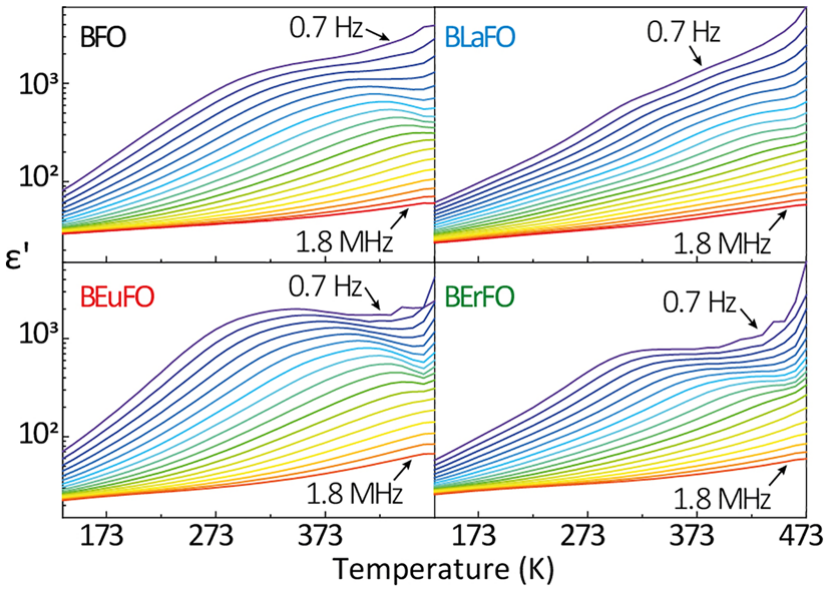
Temperature and frequency dependence of the real part of dielectric permittivity of BFO, BLaFO, BEuFO, and BErFO samples.

Temperature variation of the dielectric response of nanosized bismuth ferrites are described as rather complex and the results of the dielectric tests are presented in Fig. 4.

Following Hunpratub et al. three temperature ranges could be distinguished on the εʹ vs. T spectra in BFO, which are characterized by different relaxation processes^69^: low-temperature dielectric relaxation (LTDR) in the range of ~ 133–293 K, middle-temperature dielectric relaxation (MTDR) at ~ 293–443 K, and high-temperature relaxation (HTDR) in the temperature range above ~ 443 K. In the case of studied compounds the border of mentioned processes is not well defined and difficult to distinguish. Also assignment of these relaxation processes is not easy and disputable. Nevertheless, according to Chybczyńska et al.^70^ the LTDR is related to the effect of mixed valence of the Fe ions, the MTDR attributed to grain boundary effect and the HTDR connecting to the ordering of oxygen vacancies. The analysis of measured dielectric response values and the nature of their change indicates that the dielectric response is associated with the intrinsic heterogeneity of the polycrystalline materials^36,70^ and agrees with XRD and XPS results (Figs. S1 and 1). As mentioned before, experimental powders consist of aggregated inhomogeneous nanograins of different sizes, forms, and features of the interconnection of grains and grain boundaries. It is known, that the grain boundaries and grain interiors show different electric conductivity. For instance, the conductivity of grain/crystallite boundaries in ferrites is connected to electron hopping Fe^2+^ – e^–^ → Fe^3+^ among localized ferrous and ferric ions and significantly lower than that of the interior of the grains/crystallites^70^.

### Magnetic properties

**Figure 5:**
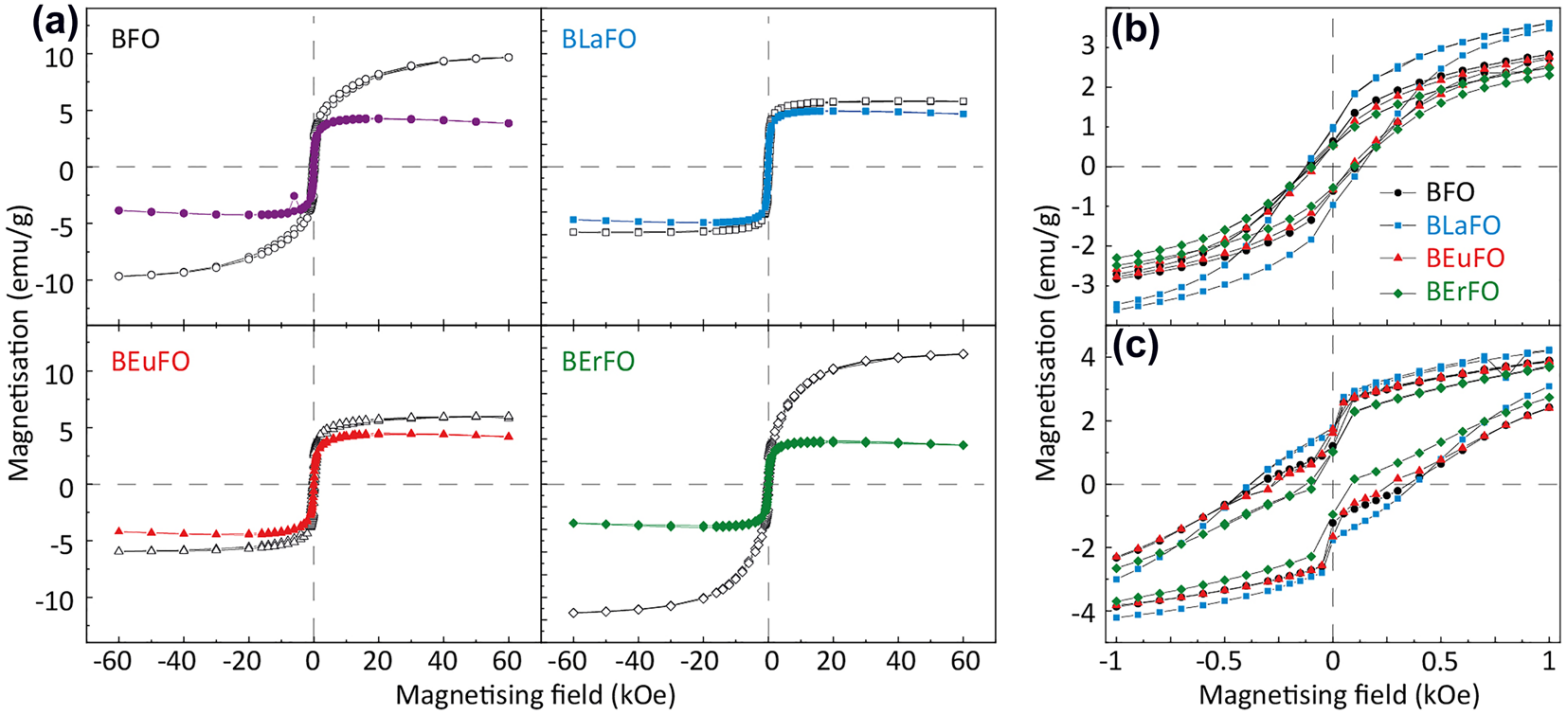
(**a**) M-H curves of neat and doped BFO, measured at 300 K (coloured—filled symbols) and 2 K (black—open symbols), (**b**) enlarged M-H curves of neat and doped BFO measured at 300.0 K and (c) 2.0 K.

Figure 5 shows magnetisation hysteresis loops of the samples at 300.0 K and 2.0 K after subtraction of the paramagnetic contribution. Despite an elaborate model for hysteresis subtraction, the calculated and subtracted linear paramagnetic component at 300.0 K seems slightly overestimated, as the corrected magnetisation decreases in high magnetic fields. The results of the data analysis show that the magnetic properties of synthesized materials were significantly influenced by rare-earth doping. In the low magnetic field regime at 300.0 K, all samples exhibited similar behavior with ferromagnetic-like hysteresis and coercivity varying from 83.24 Oe for the BEuFO sample up to 127.04 Oe for the BLaFO sample. However, coercive fields decrease with the substitution of Bi^3+^ by smaller-sized Eu^3+^ and Er^3+^ ions. Such behavior could be related to distortion and shrinkage of the BFO crystal cells^36^.

Subtraction of the paramagnetic component of M-H curves measured at 2.0 K resulted in a more reliable analysis of hysteresis, as the saturation observed in Fig. 5c is much more pronounced in the high magnetic field regime.

We found that the saturation magnetisation increases for all samples at 2.0 K in comparison with the saturation magnetisation values for the same samples at 300.0 K. The most significant increase was registered for neat BFO and Er^3+^-doped samples. The hysteresis loops obtained at 2.0 K exhibit a wasp-like shape. This can indicate that samples contain particles of a broad distribution of sizes with different values of coercivity^71^. The hysteresis loops at 2.0 K were successfully fitted using two superparamagnetic and one ferromagnetic Langevin-type curves as well as a term linear to the external magnetic field (the details described in the Supplementary Material). All calculated parameters of M-H curves are summarised in Table 4.

**Table 4:** Coercive field and the saturation magnetisation based on the fits.

Sample	Temperature (K)	Coercivity of the sample (Oe)	Coercivity of the ferromagnetic component (Oe)	Saturation magnetisation of the ferromagnetic component (emu/g)
BFO	300.0	108.19	124.3 ± 1.3	4.593 ± 0.060
	2.0	336.41	555.8 ± 7.9	10.45 ± 0.43
BLaFO	300.0	127.04	133.4 ± 2.0	5.161 ± 0.082
	2.0	381.95	487.3 ± 5.2	5.834 ± 0.092
BEuFO	300.0	83.24	107.7 ± 5.0	4.886 ± 0.085
	2.0	268.34	536.0 ± 21	6.13 ± 0.24
BErFO	300.0	98.06	115.5 ± 3.4	4.026 ± 0.084
	2.0	86.6	490.0 ± 17	12.07 ± 0.16

The observed saturation magnetisation of the neat BFO sample is notably higher than often reported in the literature. Huang et al.^8^ reported the saturation magnetisation of ferromagnetic part of the hysteresis loop of the sample containing 18 nm BFO particles to be around 0.13 emu/g at 300 K. At the same time, the authors showed that the highest saturation magnetisation of 0.15 emu/g was observed for BFO with nanoparticles of 62 nm^8^, which was ascribed to the structural anomaly emerging when the nanoparticle size approached the period of spiral-modulated spin structure. In a study by Park et al.^9^, the highest observed saturation magnetisation was estimated at 1.55 emu/g for 14 nm nanoparticles. Jayakumar et al.^72^ reported values smaller than 0.1 emu/g at 7.0 T without subtraction of significant paramagnetic components. However, there are several publications reporting saturation magnetisation in the same order of magnitude as our results. Verma and Kotnala^73^ found the saturation magnetisation at 300 K of their BFO at 4.73 emu/g, increasing up to 8.99 emu/g upon 10% doping with Pb. Tahir et al.^74^ showed the increase of room temperature saturation magnetisation from 9.202 emu/g for neat BFO to as high as 21.097 emu/g upon 30% doping with Ca.

The ZFC and FC (see Fig. S2) curves exhibit splitting that can be attributed to the coexistence of ferromagnetic and antiferromagnetic phases in the sample, a phenomenon known to happen for BiFeO_3_^8^. The shape of the registered thermomagnetic curve is similar to that reported by Huang et al.^8^ for BiFeO_3_ nanoparticles of 83 nm in diameter, but with magnetisation values higher by a factor of about 100. Notably, the local maximum in the curves, interpreted as the spin-glass freezing temperature^9^, is not present in our data.

There are a few possible reasons for such discrepancy between most of the literature data and the experimental values obtained in this work. For instance, the concentration of oxygen vacancies in the structure of the obtained BFO was reported to influence the magnetisation of Dy-doped BFO^75^. The increasing number of oxygen vacancies could lead to the overall enhancement in the magnetisation of BFO-based nanomaterials, as it promotes an increase in the amount of Fe^2+^ to Fe^3+^ ions transitions for compensation of excess positive charge. The oxygen vacancies could also lead to an increased surface spin disorder by uncompensated spins from Fe^3+^ ions. Another possible reason is that saturation magnetisation could increase due to the distortion of the crystal structure of nanograins. A decrease in crystallite size increases the influence of uncompensated surface spins with increased contribution of the uncompensated ferromagnetic surface layers to ferromagnetic components of magnetisation^9,76^. Simultaneously, smaller crystalline sizes of BFO nanomaterials are related to a suppression of spiral spin order and higher shape anisotropy. Both factors were reported to have a significant influence on the ferromagnetism of BFO-based nanomaterials^9,77^. At the same time, rare-earth doping could induce an increase in the canting angle of layers due to the tilt of FeO_6_ octahedra^78^, which even further increases the overall magnetisation. And last but not the least, magnetisation is highly sensitive to impurities and phase inhomogeneity. As it was shown previously by XRD and XPS analysis, the experimental samples contain Bi_2_Fe_4_O_9_ and Bi_2_O_3_ by-phases with a different magnetization behavior^79–81^, which form inhomogeneous structures in combination with BFO and BReFO phases. However, despite significant paramagnetic-like components, the saturation magnetisation of the ferromagnetic part of the reported hysteresis loop for Bi_2_Fe_4_O_9_ was not higher than 0.3 emu/g. Based on this fact we could conclude the presence of the Bi_2_Fe_4_O_9_ phase at the estimated concentration cannot be responsible for the observed magnetisation values. Diamagnetic bismuth(III) oxide^82^ could only add to the linear term of the hysteresis loop but would not affect saturation values. As the saturation magnetisation of Fe_3_O_4_ is estimated at 98 emu/g^83^, some impurities as low as 5% would be sufficient to explain the value of the observed saturation magnetisation. This hypothesis can be supported by the considerable share of Fe(II) species in comparison to Fe(III) species, evidenced by XPS measurements. At the same time, however, the results of XRD analysis and Mössbauer spectroscopy do not expose the presence of any significant amount of iron oxide magnetic species, regardless of whether ferromagnetic or superparamagnetic. This fact leads us to the conclusion that even if there are some other than BFO magnetic phases in the samples contributing to the overall magnetisation, they would not be a significant part of the sample mass.

## Conclusions

In this study, a series of Bi_0.9_RE_0.1_FeO_3_ (RE = La, Eu, and Er) powders was prepared by the SCS method and their structural features, and factors that influence nanomaterials electric, and magnetic properties were carefully studied and analysed.

1. The results of XRD, XPS, and Mössbauer spectroscopy analyses revealed that all obtained materials are nanocrystalline powders with predominantly BFO or modified B*RE*FO nanograins and thin outer layers enriched with Bi_2_Fe_4_O_9_, Bi_2_O_3_, and rare-earth metal oxides. The formation of an inhomogeneous structure was associated with the unequal influence of the external atmosphere and temperature gradient during synthesis and post-synthesis quenching.
2. The electrical measurements confirmed the ferroelectric behaviour of neat and RE-doped BFO with the presence of spontaneous polarization.
3. It was found, the synthesized materials are characterized with significantly improved magnetic properties, which are related to the structural transformation, promoted by the RE doping. At the same time, the analyses showed a decrease in coercive fields with the substitution of Bi^3+^ by smaller-sized Eu^3+^ and Er^3+^ ions due to the distortion of the crystal cells and volume shrinkage.
4. All the studied materials are characterized by relatively high values of magnetisation and coercivity. The highest measured values of saturation magnetisation of the ferromagnetic component (5.161 emu/g) and coercivity (133.4 Oe) among all the studied materials at 300.0 K are found for the Bi_0.9_La_0.1_FeO_3_ nanopowder. The main contribution to the magnetic behaviour of the nanopowders was associated with the BFO-based phase, as the presence of a significant amount of iron oxides or any other ferromagnetic phases were excluded by XRD and Mössbauer spectroscopy.
5. Based on the data analysis we concluded, the possible explanations for so high magnetic properties are (i) the influence of oxygen vacancies in the structure of the obtained BFO, supported by the presence of high-temperature dielectric relaxations in the broadband dielectric spectroscopy spectra; and (ii) nanosized structural parameters of crystallites, supported by narrow ferroelectric hysteresis loops.
6. The results of the study showed that SCS is a promising method for the preparation of a broad variety of neat and doped BFO-based nanomaterials with enhanced magnetic properties. Overall the SCS-obtained BFO-based materials are promising candidates for application as components of various electronic devices. The same SCS approach could also be used for the synthesis of other types of ferromagnetic nanomaterials with enhanced properties.

## Supplementary Information

Supplementary Information.
