# Supplementary material for: Structural, electrical, and magnetic study of La-, Eu-, and Er- doped bismuth ferrite nanomaterials obtained by solution combustion synthesis
Source: Sci Rep. 2021 Nov 23;11:22746. doi: 10.1038/s41598-021-01983-z (PMC8610975; doi:10.1038/s41598-021-01983-z)
Supplement: Supplementary file 1 — Supplementary Information. [file 41598_2021_1983_MOESM1_ESM.pdf]

Supporting Information

for

Structural, electrical, and magnetic study of La-, Eu-, and Er- doped bismuth ferrite nanomaterials obtained by solution combustion synthesis

Angelika Wrzesińska[a,*], **Alexander Khort**[b, c,*], **Marcin Witkowski**[d], **Jacek Szczytko**[d], **Jacek Ryl**[e] , **Jacek Gurgul**[f], **Dmitry S. Kharitonov**[f,g], **Kazimierz Łątka**[h], **Tadeusz Szumiata**[j], **Aleksandra Wypych-Puszkarz**[a]

[a]Lodz University of Technology, Zeromskiego 116, 90-924 Lodz, Poland

[b]KTH Royal Institute of Technology, Stockholm, Sweden

[c]National University of Science and Technology "MISIS", Moscow, Russia;

[d]University of Warsaw, Pasteura 1, 02-093 Warsaw, Poland

[e]Gdańsk University of Technology, 11/12 Narutowicza st., 80-233, Gdansk, Poland

[f]Jerzy Haber Institute of Catalysis and Surface Chemistry, Polish Academy of Sciences, Niezapominajek 8, PL-30239 Krakow, Poland

[g]Research and Development Center of Technology for Industry, Ludwika Warynskiego 3A, PL–00645 Warsaw, Poland

[h]Marian Smoluchowski Institute of Physics, Jagiellonian University, Łojasiewicza 11, 30-348 Kraków, Poland

[i]Kazimierz Pulaski University of Technology and Humanities in Radom, Stasieckiego Str. 54, 26-600 Radom, Poland

SI. 1. X-ray diffraction patterns

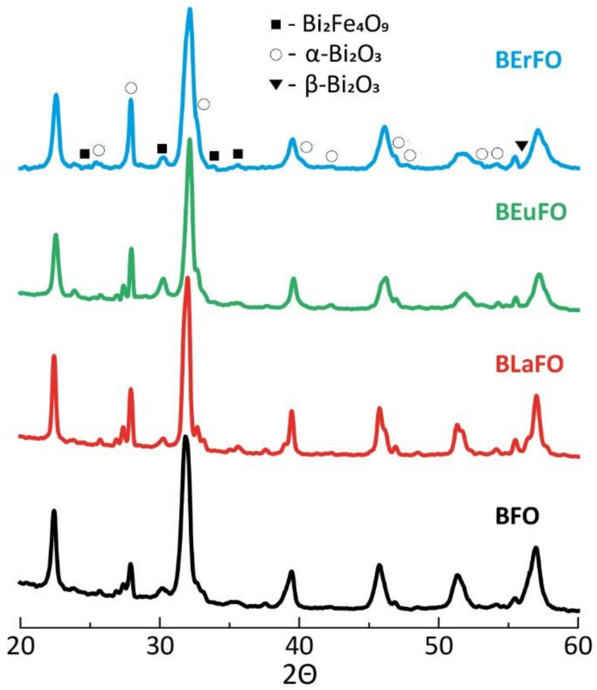

Figure S1. Results of XRD analysis of BFO, BLaFO, BEuFO, and BErFO samples.

SI. 2. Deconvolution of hysteresis loops into components

To increase the reliability of the fitting procedure, the points registered in the lower part of the hysteresis loops were flipped into the upper part of respective loops by symmetrical rotation about the (0,0) point of the $M(H)$ dependence. The points representing both halves were corresponding to each other reasonably well.

Magnetization was modeled using Langevin functions with the parameter a of a form:

$$\mathcal{L}_a(x) = \coth ax - \frac{1}{ax}$$

At 300.0 K one branch of all the hysteresis loops was well explained by the fit of the following equation:

$$M(H) = aH + M_{S,1}\mathcal{L}_{a_1}(H) + M_{S,2}\mathcal{L}_{a_2}(H - H_C)$$

where: $M(H)$ is the observed magnetization of the hysteresis loop, H is the magnetic field strength, a is the linear term representing the sum of diamagnetic and paramagnetic components to the magnetization, $M_{S,1}$ and $M_{S,2}$ are the saturation magnetizations of the components represented by the Langevin curves, $\mathcal{L}_{a_1}(H)$ is the superparamagnetic-like component, H_C is the coercivity of the ferromagnetic component (which represents the coercivity of the sample decently as well) and $\mathcal{L}_{a_2}(H - H_C)$ represents the ferromagnetic component in the overall magnetization. $M_{S,1}$, $M_{S,2}$, a, a_1, a_2 and H_C were the parameters optimized during the fitting procedure.

At 2.0 K the function fitted to the experimental data was similar, although having one more component:

$$M(H) = aH + M_{S,1}\mathcal{L}_{a_1}(H) + M_{S,2}\mathcal{L}_{a_2}(H - H_C) + M_{S,3}\mathcal{L}_{a_3}(H)$$

where symbols are the same as for 300.0 K, but $M_{S,3}$ is the saturation magnetization of the third Langevin-type component, represented by $\mathcal{L}_{a_3}(H)$. Eight parameters were optimized during the fitting procedure, namely: $M_{S,1}$, $M_{S,2}$, $M_{S,3}$, a, a_1, a_2, a_3 and H_C. This function was sufficient to reliably deconvolute the hysteresis, however for the BLaFO sample the a_3 was diverging into very high values, resulting in numerical errors rendering the reliable analysis impossible to obtain. Considering the behavior of the Langevin function as the a parameter diverges, for the BLaFO sample an alternative dependence was used:

$$M(H) = aH + M_{S,1}\mathcal{L}_{a_1}(H) + M_{S,2}\mathcal{L}_{a_2}(H - H_C) + M_{S,3}(2\,\theta(H) - 1)$$

where $\theta(H)$ is the Heaviside step function. Such a definition allows the interpretation of $M_{S,3}$ consistent with other samples.

All the fitting was performed using the NonlinearModelFit function of the Wolfram Language, executed by the Wolfram Mathematica 10.3 software package. Experimental points were used

with weights defined as the inverse square of the uncertainty of a particular point. Despite a considerable number of parameters optimized during the procedure, there were more than 100 experimental data points used for the fit, decreasing the risk of overfitting.

SI. 3. Temperature-dependent measurements of magnetic properties

ZFC/FC curves of the neat BFO and RE doped samples are presented in Figure S2.

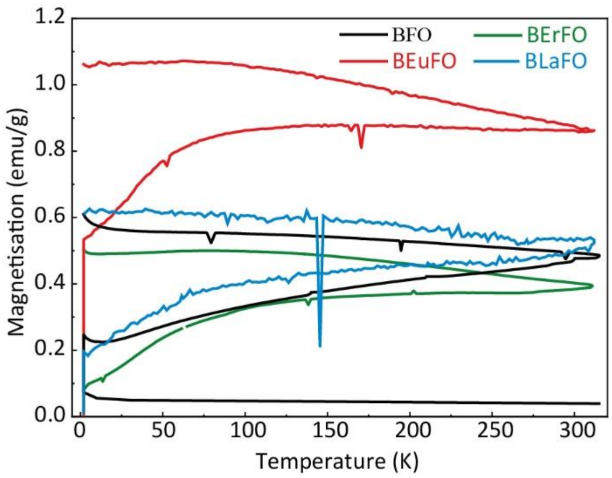

Figure S2. ZFC/FC curves of neat and substituted BFO measured at 100 Oe.
